# The Impact of Serial Remote Ischemic Conditioning on Dynamic Cerebral Autoregulation and Brain Injury Related Biomarkers

**DOI:** 10.3389/fphys.2022.835173

**Published:** 2022-02-22

**Authors:** Yang Qu, Peng Zhang, Qian-Yan He, Ying-Ying Sun, Mei-Qi Wang, Jia Liu, Pan-Deng Zhang, Yi Yang, Zhen-Ni Guo

**Affiliations:** ^1^Stroke Center & Clinical Trial and Research Center for Stroke, Department of Neurology, The First Hospital of Jilin University, Changchun, China; ^2^China National Comprehensive Stroke Center, Changchun, China; ^3^Jilin Provincial Key Laboratory of Cerebrovascular Disease, Changchun, China; ^4^Shenzhen Institutes of Advanced Technology, Chinese Academy of Sciences, Shenzhen, China

**Keywords:** remote ischemic conditioning, dynamic cerebral autoregulation, biomarkers, intervention, transfer function analysis, vascular function

## Abstract

**Objective:**

Recent studies have demonstrated the positive roles of remote ischemic conditioning (RIC) in patients with cerebrovascular diseases; however, the mechanisms remain unclear. This study aimed to explore the effect of serial RIC on dynamic cerebral autoregulation (dCA) and serum biomarkers associated with brain injury, both of which are related to the prognosis of cerebrovascular disease.

**Methods:**

This was a self-controlled interventional study in healthy adults. The RIC was conducted twice a day for 7 consecutive days (d1–d7) and comprised 4 × 5-min single arm cuff inflation/deflation cycles at 200 mmHg. All participants underwent assessments of dCA ten times, including baseline, d1, d2, d4, d7, d8, d10, d14, d21, and d35 of the study. Blood samples were collected four times (baseline, d1, d7, and d8) immediately after dCA measurements. The transfer function parameters [phase difference (PD) and gain] were used to quantify dCA. Four serum biomarkers associated with brain injury, ubiquitin C-terminal hydrolase-L1, neuron-specific enolase, glial fibrillary acidic protein, and S100β were tested.

**Results:**

Twenty-two healthy adult volunteers (mean age 25.73 ± 1.78 years, 3 men [13.6%], all Asian) were enrolled in this study. Bilateral PD values were significantly higher since four times of RIC were completed (d2) compared with PD values at baseline (left: 53.31 ± 10.53 vs. 45.87 ± 13.02 degree, *p* = 0.015; right: 54.90 ± 10.46 vs. 45.96 ± 10.77 degree, *p* = 0.005). After completing 7 days of RIC, the significant increase in dCA was sustained for at least 28 days (d35, left: 53.11 ± 14.51 degree, *P* = 0.038; right: 56.95 ± 14.57 degree, *p* < 0.001). No difference was found in terms of different serum biomarkers related to brain injury before and after RIC.

**Conclusion:**

The elevation in dCA was detected immediately after four repeated times of RIC, and 7-day consecutive RIC induced a sustained increase in dCA for at least 28 days and did not affect blood biomarkers of brain injury in healthy adults. These results will help us to formulate detailed strategies for the safe and effective application of RIC in patients with cerebrovascular disease.

## Introduction

Remote ischemic conditioning (RIC) refers to an intervention that offers remote tissues and organs a resistance capacity to ischemia/reperfusion injury through small doses of reversible episodes of ischemia and reperfusion (Hess et al., 2015; Heusch et al., 2015) that activate neurogenic pathways, humoral factors, and the immune system (Anrather and Hallenbeck, 2013). Recent studies have demonstrated that RIC could increase cerebral perfusion, promote hematoma resolution, facilitate recovery of nerve function, and improve the clinical prognosis of patients with cerebrovascular diseases (Meng et al., 2012; An et al., 2020; Zhao et al., 2021). However, the mechanism is not fully elucidated. Our previous study found that dynamic cerebral autoregulation (dCA), an important indicator of cerebrovascular function which related to the prognosis of cerebrovascular disease (Xiong et al., 2017) was improved after once round of RIC (Guo et al., 2019); however, the exact effect of serial RIC on dCA was unclear. Additionally, the dCA alteration period after serial RIC remains unknown. Solving these problems will help us to formulate detailed strategies for the safe and effective application of RIC in patients with cerebrovascular disease.

Brain injury related biomarkers concerning neuronal cell body injury [ubiquitin C-terminal hydrolase-L1 (UCH-L1) and neuron-specific enolase (NSE)] and astroglial injury [glial fibrillary acidic protein (GFAP) and S100β] can reflect astrocyte activation, neuronal damage, and impairment of the blood-brain-barrier’s integrity in patients with cerebrovascular disease (Day and Thompson, 2010; Zhao et al., 2017; Wang et al., 2018; Michetti et al., 2019; Li et al., 2020) and are correlated with the infarct size, neurological functional status, and clinical outcomes (Bharosay et al., 2012; Wang et al., 2017; Lasek-Bal et al., 2019; Luger et al., 2020; Onatsu et al., 2020). Whether RIC can affect brain injury related biomarkers in animal models was controversial (Herajarvi et al., 2017; Sandweiss et al., 2017; Ren et al., 2018), and until very recently, there was a lack of human studies.

Hence, the first objective of this study was to observe the exact impact of serial RIC on dCA. This included determining when dCA was increased during serial RIC and the duration of the effect of serial RIC. The second was to explore changes of brain injury related biomarkers after serial RIC, including UCH-L1, NSE, GFAP, and S100β.

## Materials and Methods

### Participants

Thirty-six healthy volunteers were recruited between May and June 2021. The inclusion criteria were as follows: (1) age 18–50 years (men and women, Asian) and (2) willing to participate in follow-up visits. The exclusion criteria were as follows: (1) current or a history of chronic physical diseases or mental diseases (including hypertension, diabetes mellitus, generalized anxiety disorder, depression, insomnia, and chronic heart disease), (2) having infectious diseases in the past month, (3) pregnant or lactating (women), (4) engaged in smoking or heavy drinking (formerly or currently), and (5) inability to cooperate sufficiently to complete the dCA examination (e.g., due to a condition, such as atrial fibrillation) during the recording. All participants signed a written informed consent form before participating in the study.

### Study Design

**FIGURE 1:**
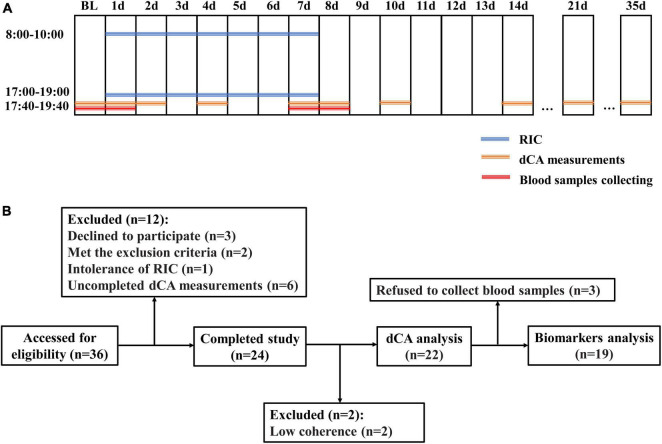
**(A)** Protocols of the study. **(B)** Flowcharts of the study. BL, baseline; RIC, remote ischemic conditioning; and dCA, dynamic cerebral autoregulation.

This was a self-controlled interventional study. The dCA of each participant was measured at 17:40–19:40 at the beginning of the study (baseline). The RIC was performed twice daily, 8:00–10:00 and 17:00–19:00, for 7 days (d1–d7). To observe the time when dCA was increased during serial RIC, dCA was measured at d1, d2, d4, and d7 immediately after the second RIC treatment of each day. To determine the duration of the effect of serial RIC, dCA was measured at 17:40–19:40 immediately, 1 day, 3 days, 7 days, 14 days, and 28 days after RIC (d7, d8, d10, d14, d21, and d35, respectively). The protocol is shown in Figure 1A. This study was registered at ClinicalTrials.gov (NCT04899362) and approved by the Ethics Committee of the First Hospital of Jilin University.

### Intervention

The RIC was performed on each volunteer using an automatic medical device (BB-RIC-D1/LAPUL Medical Devices Co., Ltd., Shanghai, China). Each RIC session comprised inflating one arm cuff to 200 mmHg pressure for 5 min followed by a 5-min reperfusion period, repeated for four cycles for a total of 40 min.

### Dynamic Cerebral Autoregulation Measurement and Analysis

To minimize confounding stimuli, all dCA measurements were performed in a quiet examination room with a controlled temperature ranging from 20 to 24°C. Before the examination, participants were instructed to relax in a supine position for 10 min. Baseline blood pressure and heart rate were then measured at the left brachial artery using an automatic blood pressure monitor (Omron 711). Cerebral blood flow velocity was measured using a transcranial Doppler (MultiDop X2, DWL, Sipplingen, Germany). Two 2-MHz probes were fixed with a customized head frame at the bilateral temporal bone window at a depth of 45–60 mm. Spontaneous arterial blood pressure was recorded non-invasively and simultaneously by a servo-controlled plethysmograph (Finometer model 1, FMS, Rotterdam, Netherlands) on the middle finger. Real-time recording of cerebral blood flow velocity and arterial blood pressure lasted for 10 min and was stored for further analysis.

Data on cerebral blood flow velocity and arterial blood pressure were processed using MATLAB (MathWorks, Inc., Natick, MA, United states) using scripts developed by the research team, as previously reported (Ma et al., 2016; Guo et al., 2019). In each participant, the two signals were aligned using a cross-correlation function. A third-order Butterworth low-pass filter with a cutoff frequency of 0.5 Hz was applied, and the signals were down-sampled to 1 Hz. Phase difference (PD), gain, and coherence function within a low-frequency range, 0.06–0.12 Hz, were then derived from transfer function analysis to assess the dynamic association between cerebral blood flow velocity and arterial blood pressure. PD with low coherence (≤0.40) was not available for further statistical analysis.

### Blood Samples and Serum Testing

Blood samples were drawn from the cubital vein of each participant at baseline, d1, d7, and d8 immediately after the dCA measurements (Figure 1A). These samples were centrifuged immediately after collection, and the serum were stored at −80°C until batch evaluation.

The MS-Fast/Aceso 80A automated magnetic particle-based chemiluminescent enzyme immunoassay analyzing system developed by Sophonix was used to test UCH-L1, NSE, GFAP, and S100β levels. Biotinylated capture antibody connected with various proteins and alkaline phosphatase labeled detection antibodies in a sandwich-type detection manner. This immune complex was further reacted with excessive streptavidin-coated magnetic particles to form a complex. In a magnetic field, the complex was enriched, and the sensitivity was thus enhanced. The limit of detection of this method was 0.8 pg/mL. The coefficient of variation was <8.0%.

### Statistical Analyses

The data were analyzed using the Statistical Program for Social Sciences version 26.0 (SPSS; IBM, West Grove, PA, United States). The distribution of data was assessed using a one-sample Kolmogorov–Smirnov test. Normally distributed continuous variables are shown as mean ± standard deviation, and non-normally distributed data are presented as the median and interquartile range. Repeated measurement analysis of variance was used to test the differences in the observed dCA values on different days. Two general linear models were used for the repeated measurements. To observe when dCA was increased during serial RIC, dCA parameters at baseline, d1, d2, d4, and d7 were included. To assess the duration of the effect of serial RIC, baseline, d7, d8, d10, d14, d21, and d35 were included. To compare the difference between the baseline and other days, a paired *t*-test was used. The changes in blood biomarker levels were analyzed by Friedman test. Baseline, d1 and d7, and baseline, d7 and d8 were compared, respectively. A two-tailed *p*-value < 0.05 was considered statistically significant.

## Results

### Participant Characteristics

Thirty-six healthy adult volunteers were assessed for eligibility, and six participants who declined to participate, met the exclusion criteria, or were intolerant to RIC were excluded. Eight participants were excluded because of incomplete dCA measurements and low coherence. Finally, 22 healthy adult volunteers (mean age 25.73 ± 1.78 years, 3 men [13.6%], all Asian, Figure 1B) were enrolled in this study. The clinical characteristics of the participants are presented in Table 1.

**TABLE 1:** Clinical characteristics of study participants.

Variables	Total (*n* = 22)
Gender (male)	3 (13.6%)
Age (year)	25.73 ± 1.78
Height (cm)	164.50 ± 8.36
Weight (kg)	56.81 ± 9.24
BMI (kg/m2)	20.88 ± 2.10
Waist (cm)	71.13 ± 7.36
Hip (cm)	89.72 ± 8.89
Waist/hip ratio	0.79 ± 0.61
Baseline systolic blood pressure (mmHg)	106.45 ± 6.22
Baseline diastolic blood pressure (mmHg)	62.50 ± 6.38
Baseline heart rate (beats/min)	73.82 ± 7.04
Baseline left systolic cerebral blood flow velocity (cm/s)	107.86 ± 16.18
Baseline left diastolic cerebral blood flow velocity (cm/s)	55.64 ± 7.72
Baseline right systolic cerebral blood flow velocity (cm/s)	110.32 ± 13.84
Baseline right diastolic cerebral blood flow velocity (cm/s)	56.59 ± 6.77

### Dynamic Cerebral Autoregulation During Serial Remote Ischemic Conditioning

**FIGURE 2:**
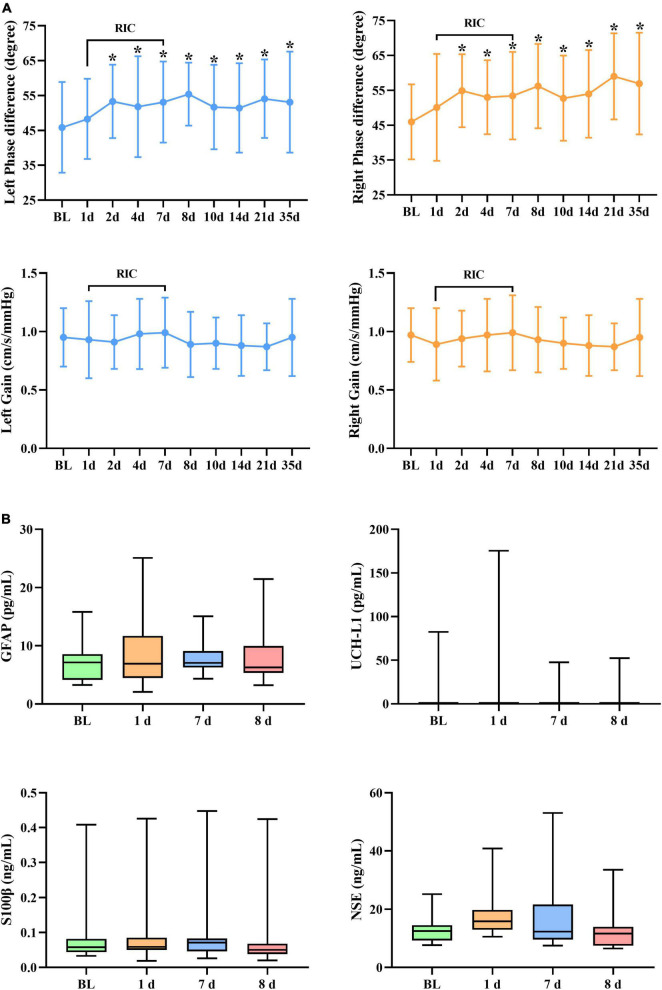
**(A)** Dynamic cerebral autoregulation (dCA) parameters and **(B)** serum biomarkers during and after RIC. RIC, remote ischemic conditioning; dCA, dynamic cerebral autoregulation; GFAP, glial fibrillary acidic protein; UCH-L1, ubiquitin C-terminal hydrolase-L1; and NSE, neuron-specific enolase. *p < 0.05 compared with baseline.

A summary of repeated measurements for dCA parameters across different days during RIC is presented in Table 2. The general linear model identified the highly significant effects of RIC on both left (*p* = 0.033) and right (*p* = 0.017) PD, but not on gain. A comparison of bilateral PD and gain between the RIC and baseline is shown in Figure 2A and Table 3. PD values were significantly higher since four times of RIC were completed compared with PD values at baseline (left: 53.31 ± 10.53 vs. 45.87 ± 13.02 degree, *p* = 0.015; right: 54.90 ± 10.46 vs. 45.96 ± 10.77 degree, *p* = 0.005). However, if RIC was repeated twice, both left and right PD values revealed no difference (48.29 ± 11.53 degree, *p* = 0.366 and 50.09 ± 15.34 degree, *p* = 0.366, respectively).

**TABLE 2:** Summary of repeated measurements for dynamic cerebral autoregulation (dCA) parameters across different days during remote ischemic conditioning (RIC).

Total (*n* = 22)	*F*	*p*
Left phase difference (degree)	2.752	0.033
Right phase difference (degree)	3.990	0.017
Left gain (cm/s/mmHg)	0.524	0.720
Right gain (cm/s/mmHg)	0.722	0.588

*dCA, dynamic cerebral autoregulation; RIC, remote ischemic conditioning.*

**TABLE 3:** DCA parameters in participants during RIC.

	Baseline	1 d/twice RIC completed	2 d/four times RIC completed	4 d/8 times RIC completed	7 d/14 times RIC completed
Left phase difference (degree)	45.87 ± 13.02	48.29 ± 11.53	53.31 ± 10.53	51.80 ± 14.47	53.11 ± 11.64
t		−0.923	−2.637	−2.145	−2.418
p		0.366	0.015	0.044	0.025
Right phase difference (degree)	45.96 ± 10.77	50.09 ± 15.34	54.90 ± 10.46	53.03 ± 10.61	53.45 ± 12.55
t		−1.371	−3.125	−2.811	−3.126
p		0.185	0.005	0.010	0.005
Left gain (cm/s/mmHg)	0.95 ± 0.25	0.93 ± 0.33	0.91 ± 0.23	0.98 ± 0.30	0.99 ± 0.30
t		0.331	0.914	−0.444	−0.647
p		0.744	0.371	0.661	0.524
Right gain (cm/s/mmHg)	0.97 ± 0.23	0.89 ± 0.31	0.94 ± 0.24	0.97 ± 0.31	0.99 ± 0.32
t		1.181	0.445	−0.075	−0.511
p		0.251	0.661	0.941	0.615

*dCA, dynamic cerebral autoregulation; RIC, remote ischemic conditioning.*

### Dynamic Cerebral Autoregulation After Serial Remote Ischemic Conditioning

Figure 2A and Tables 4, 5 show the effect of RIC on bilateral PD (left: *p* = 0.021, right: *p* < 0.001). When compared with baseline values, both sides of PD were improved immediately after 7-day RIC was completed (left: 53.11 ± 11.64 vs. 45.87 ± 13.02 degree, *p* = 0.025; right: 53.45 ± 12.55 vs. 45.96 ± 10.77 degree, *p* = 0.005), and the increase was sustained for at least 28 days (left: 53.11 ± 14.51 degree, *p* = 0.038; right: 56.95 ± 14.57 degree, *p* < 0.001). The values of gain remained insignificant throughout the study.

**TABLE 4:** Summary of repeated measurements for dCA parameters across different days after RIC.

Total (*n* = 22)	*F*	*p*
Left phase difference (degree)	2.598	0.021
Right phase difference (degree)	4.475	<0.001
Left gain (cm/s/mmHg)	0.694	0.655
Right gain (cm/s/mmHg)	1.085	0.375

*dCA, dynamic cerebral autoregulation; RIC, remote ischemic conditioning.*

**TABLE 5:** DCA parameters in participants after RIC.

	Baseline	7 d/immediately after RIC	8 d/1 day after RIC	10 d/3 days after RIC	14 d/7 days after RIC	21 d/14 days after RIC	35 d/28 days after RIC
Left phase difference (degree)	45.87 ± 13.02	53.11 ± 11.64	55.41 ± 9.01	51.70 ± 12.12	51.45 ± 12.84	54.07 ± 11.27	53.11 ± 14.51
t		−2.418	−3.999	−2.194	−2.498	−2.561	−2.208
p		0.025	<0.001	0.040	0.021	0.018	0.038
Right phase difference (degree)	45.96 ± 10.77	53.45 ± 12.55	56.22 ± 12.08	52.74 ± 12.23	53.99 ± 12.59	59.02 ± 12.32	56.95 ± 14.57
t		−3.126	−4.124	−2.540	−3.370	−5.399	−3.856
p		0.005	<0.001	0.019	0.003	<0.001	<0.001
Left gain (cm/s/mmHg)	0.95 ± 0.25	0.99 ± 0.30	0.89 ± 0.28	0.94 ± 0.22	0.92 ± 0.24	0.87 ± 0.28	0.97 ± 0.27
t		−0.647	1.070	0.398	0.510	1.564	−0.237
p		0.524	0.297	0.695	0.615	0.133	0.815
Right gain (cm/s/mmHg)	0.97 ± 0.23	0.99 ± 0.32	0.93 ± 0.28	0.90 ± 0.22	0.88 ± 0.26	0.87 ± 0.20	0.95 ± 0.33
t		−0.511	0.579	1.382	1.386	1.877	0.177
p		0.615	0.569	0.182	0.180	0.074	0.851

*dCA, dynamic cerebral autoregulation; RIC, remote ischemic conditioning.*

### Serum Biomarkers

No significant difference was found in terms of UCH-L1, NSE, GFAP, and S100β levels (Figure 2B).

## Discussion

In this study, the elevation of dCA was detected immediately after four repeated times of RIC in healthy adults, and after completing 14 times of RIC in 7 consecutive days, the elevation of dCA lasted for at least 28 days. We did not detect any effects of RIC on serum biomarkers of brain injury, suggesting that RIC is safe. These results will help us to formulate detailed strategies for the safe and effective application of RIC in patients with cerebrovascular disease.

To date, few studies have assessed the effect of RIC on dCA in humans. A previous study has performed RIC once on bilateral arms and showed that a single bout of RIC did not acutely influence dCA in either healthy individuals or those with increased cardiovascular risk (Carter et al., 2020). Our previous study has shown that RIC performed only once on one arm and one thigh did not induce an immediate increase in dCA (Guo et al., 2019). The results of another study have further indicated that only a single session of RIC, although performed on dual thighs, was insufficient to evoke changes in not only cerebrovascular function but also peripheral and pulmonary vascular function in healthy adults (Rieger et al., 2017). These previous studies have consistently found that single RIC treatments do not provide immediate benefits to cerebrovascular function, regardless of the RIC strategy. This study further showed that performing RIC twice was still ineffective in increasing dCA immediately.

It is worth noting that RIC-mediated protection is known to be triphasic in nature. There is a rapid protective period that is achieved immediately or within minutes and subsides within a few hours of application, an intermediate protective function from 12 h after RIC, and a more prolonged, delayed protective window (1–3 days) (Ren et al., 2008; Durukan and Tatlisumak, 2010; Koch et al., 2014). However, as mentioned above, the existing studies have concluded that the effect of RIC on cerebral measures was not observed immediately; one possible explanation is that the cerebral protective function was not activated within the initial protective phase and possibly started in an earlier intermediate tolerance (Guo et al., 2019). Interestingly, in our study, dCA increased after performing four repeated times of RIC. There were two possible reasons for this: one was that dCA was increased because of the superimposed effect of four serial 4 times of RIC, and the other was that a delayed cerebral protective function was activated. The superimposed effect and delayed activation may also interact to promote dCA improvement.

Additionally, our study suggested a prolonged effect of 7-day RIC on dCA improvement, which could last at least 28 days. To the best of our knowledge, this was the longest follow-up taken of functional measurements of cerebrovascular after RIC. A previous study conducted a randomized pilot study to explore whether daily RIC for 7 days could improve endothelial and cerebrovascular function in patients with type 2 diabetes mellitus. They showed that cerebrovascular function assessed by dCA remained unchanged (Maxwell et al., 2019). Evidence from both animal models and clinical studies has suggested that diabetes may abolish the protective effect of RIC (Jensen et al., 2013; Baranyai et al., 2015; Hu et al., 2017) potentially because of impairment in the neurogenic pathways thought to potentiate the action of RIC (Tyagi et al., 2019). Therefore, the negative effect of 7-day RIC on dCA in patients with diabetes mellitus might be due to diabetic neuropathy. In healthy young adults, previous studies have evaluated the effect of RIC repeated over 7 consecutive days on human microcirculation and showed that cutaneous vascular function was improved and remained elevated 7–8 days after RIC (Jones et al., 2014; Lang et al., 2019). The sustained positive effect of RIC on microvasculature was consistent with our findings in dCA. Moreover, our study indicated a significantly longer protective effect.

Ubiquitin C-terminal hydrolase-L1 and NSE are neuronal cell body injury markers, the former mainly resides in neuronal cell body cytoplasm, and the latter exists as a homodimer in mature neurons and neuroendocrine cells. GFAP and S100β are predominantly expressed in astrocytes as cytoskeletal and calcium-binding protein, respectively, reflecting astroglial injury (Wang et al., 2018). The elevation of these markers in serum could be detected several hours after brain injury (Bharosay et al., 2012; Lasek-Bal et al., 2019; Michetti et al., 2019; Luger et al., 2020; Onatsu et al., 2020). The sensitivity of the combination of UCH-L1 and GFAP in the diagnosis of brain injury was even higher than computed tomography imaging and had already been approved by the United States Food and Drug Administration (Bazarian et al., 2018; Luger et al., 2020). Our study did not discover any significant changes of UCH-L1, NSE, GFAP, and S100β, suggesting that serial RIC did not induce any brain injury or damage the integrity of the blood-brain barrier, consistent with a previous study among patients with severe carotid artery stenosis (Zhao et al., 2017). Additionally, the majority of participants tolerated the serial RIC. Thus, we consider RIC to be a safe, low-cost and easy-to-use strategy to improve dCA function.

There is now good evidence for RIC as an effective adjunctive treatment in the cardiovascular field although the protection mechanism mediated by the RIC still remains not completely known at the moment (Ho et al., 2020; Guo et al., 2021; Lukhna et al., 2021). However, for cerebrovascular diseases, there is varying level of evidence. Recently, extracellular vesicles have been thought to facilitate the transfer function between remote tissues and protected organs of RIC. Endothelial derived extracellular vesicles can further mediate vascular endothelial growth factor and endothelial nitric oxide synthase (Femmino et al., 2021; Pearce et al., 2021) both of which are major regulators of dCA (Giannopoulos et al., 2012; Guo et al., 2019). Moreover, the close association between abnormal dCA and cerebrovascular diseases has been well documented (Xiong et al., 2017). Previous studies have also proven that dCA could be considered a therapeutic target to ameliorate prognosis (Rasulo et al., 2012; Weigl et al., 2016; Minhas et al., 2020). Therefore, our study provides evidence that the serial application of RIC may result in a prolonged protection and could favorably influence the prognosis of cerebrovascular diseases.

This study has some limitations. First, to unify dCA measurement time in our study, the immediate dCA function after three times of RIC was not measured. Therefore, the alteration of dCA after three times of RIC remains unknown. Second, our study only lasted for 35 days in each participant, and the degradation of the improvements after serial 7-day RIC was not monitored. Third, this was a relatively small sample size study in healthy adults, and our findings warrant further large-scale investigations in patients with various diseases.

## Conclusion

Overall, the elevation of dCA was detected immediately after four repeated times of RIC, and 7-day consecutive RIC could elevate dCA for at least 28 days and had no effect on blood biomarkers of brain injury in healthy adults. These results will help us to formulate detailed strategies for the safe and effective application of RIC in patients with cerebrovascular disease.

## Data Availability Statement

The raw data supporting the conclusions of this article will be made available by the authors, without undue reservation.

## Ethics Statement

The studies involving human participants were reviewed and approved by the Ethics Committee of the First Hospital of Jilin University. The patients/participants provided their written informed consent to participate in this study.

## Author Contributions

Z-NG and YY contributed to the conception and design of the research. YQ, Q-YH, Y-YS, and M-QW collected the data and drafted the manuscript. PZ, JL, and P-DZ analyzed the data. YQ, Q-YH, and PZ interpreted the data. All authors contributed to the article and approved the submitted version.

## Conflict of Interest

The authors declare that the research was conducted in the absence of any commercial or financial relationships that could be construed as a potential conflict of interest.

## Publisher’s Note

All claims expressed in this article are solely those of the authors and do not necessarily represent those of their affiliated organizations, or those of the publisher, the editors and the reviewers. Any product that may be evaluated in this article, or claim that may be made by its manufacturer, is not guaranteed or endorsed by the publisher.
